# Exploring adults’ experiences of sedentary behaviour and participation in non-workplace interventions designed to reduce sedentary behaviour: a thematic synthesis of qualitative studies

**DOI:** 10.1186/s12889-019-7365-1

**Published:** 2019-08-13

**Authors:** G. H. Rawlings, R. K. Williams, D. J. Clarke, C. English, C. Fitzsimons, I. Holloway, R. Lawton, G. Mead, A. Patel, A. Forster

**Affiliations:** 1 Department of Clinical Psychology, University of Sheffield, Sheffield, UK; 2 Academic Unit of Elderly Care and Rehabilitation, Leeds Institute of Health Sciences, Temple Bank House, University of Leeds, Bradford Royal Infirmary, Bradford, BD9 6RJ UK; 3 School of Health Sciences and Priority Research Centre for Stroke and Brain Injury, University of Newcastle, Newcastle, Australia; 4 Leeds Institute of Clinical Trials Research, University of Leeds, Leeds, UK; 5 Physical Activity for Health Research Centre, University of Edinburgh, Edinburgh, UK; 6 School of Psychology, University of Leeds, Leeds, UK; 7 Geriatric Medicine, Division of Health Sciences, Centre for Clinical Brain Sciences, University of Edinburgh, Edinburgh, UK; 8 Anita Patel Health Economics Consulting Ltd, London, UK

**Keywords:** Sedentary behaviour, Sitting, Qualitative research, Physical activity, Thematic synthesis

## Abstract

**Background:**

Sedentary behaviour is any waking behaviour characterised by an energy expenditure of ≤1.5 metabolic equivalent of task while in a sitting or reclining posture. Prolonged bouts of sedentary behaviour have been associated with negative health outcomes in all age groups. We examined qualitative research investigating perceptions and experiences of sedentary behaviour and of participation in non-workplace interventions designed to reduce sedentary behaviour in adult populations.

**Method:**

A systematic search of seven databases (MEDLINE, AMED, Cochrane, PsychINFO, SPORTDiscus, CINAHL and Web of Science) was conducted in September 2017. Studies were assessed for methodological quality and a thematic synthesis was conducted. Prospero database ID: CRD42017083436.

**Results:**

Thirty individual studies capturing the experiences of 918 individuals were included. Eleven studies examined experiences and/or perceptions of sedentary behaviour in older adults (typically ≥60 years); ten studies focused on sedentary behaviour in people experiencing a clinical condition, four explored influences on sedentary behaviour in adults living in socio-economically disadvantaged communities, two examined university students’ experiences of sedentary behaviour, two on those of working-age adults, and one focused on cultural influences on sedentary behaviour. Three analytical themes were identified: 1) the impact of different life stages on sedentary behaviour 2) lifestyle factors influencing sedentary behaviour and 3) barriers and facilitators to changing sedentary behaviour.

**Conclusions:**

Sedentary behaviour is multifaceted and influenced by a complex interaction between individual, environmental and socio-cultural factors. Micro and macro pressures are experienced at different life stages and in the context of illness; these shape individuals’ beliefs and behaviour related to sedentariness. Knowledge of sedentary behaviour and the associated health consequences appears limited in adult populations, therefore there is a need for provision of accessible information about ways in which sedentary behaviour reduction can be integrated in people’s daily lives. Interventions targeting a reduction in sedentary behaviour need to consider the multiple influences on sedentariness when designing and implementing interventions.

**Electronic supplementary material:**

The online version of this article (10.1186/s12889-019-7365-1) contains supplementary material, which is available to authorized users.

## Introduction

Over the last decade, sedentary behaviour has emerged as an important public health issue. Sedentary behaviour has become the focus of research, clinical and policy interest. Evidence supporting the detrimental effects of prolonged sedentary time on health and wellbeing in individuals of all ages is rapidly growing [1–3]. In the general population, sedentary behaviour has been associated with an increased risk of a range of health problems including, cardiovascular conditions [4], mood disorders [5] and all-cause mortality [6]. Some clinical populations, for example stroke survivors [7] or those living with frailty [8], are more prone to engage in long, uninterrupted bouts of sedentariness. This is likely to contribute to increased risk of adverse health outcomes and limit the potential of rehabilitation therapies.

Sedentary behaviour is defined as ‘any waking behaviour characterised by an energy expenditure of ≤1.5 metabolic equivalents of task while in a sitting, reclining or lying posture’ (p.5). Sedentary behaviour is distinct from physical inactivity, which is defined as insufficient physical activity levels to meet current recommendations (150 min of moderate - vigorous physical activity a week) [9]. Previous systematic reviews related to sedentary behaviour have primarily focused on measurement of, determinants of and the health-related effects of sedentary behaviour, focused on interventions designed to reduce sedentary behaviour [10–12] or on whether physical activity is effective for offsetting the negative effects of sedentary behaviour [13]. These reviews explore intrapersonal, social, environmental, physical environmental and policy correlates of sedentary behaviour [14], and the relationship between sedentary behaviour and health-related quality of life. Systematic reviews of qualitative data are becoming more commonplace and have explored adults’ experiences of physical activity [15] and acceptability of physical activity interventions [16]. Qualitative research can contribute to our understanding of factors that influence sedentary behaviour, assist with identification of modifiable determinants, and help identify barriers and facilitators to promoting sedentary behaviour change.

The aims of the current review were to produce a systematic, thematic synthesis of qualitative research investigating (i) adults’ experiences of sedentary behaviour, and (ii) participation in interventions designed to reduce sedentary behaviour in adults. We sought to understand peoples’ perceptions and experiences of sedentary behaviour in order to identify what barriers and facilitators influence sedentary behaviour in adults. As this review was undertaken as part of a research programme that will develop and test a community-based sedentary behaviour reduction intervention for stroke survivors, we excluded workplace-based studies.

We also aimed explore the views of carers, relatives and health and social care professionals in relation to sedentary behaviour in adults, however, we were not able to identify any data to directly address this aim.

## Methods

### Search strategy

This review has been undertaken in line with the Preferred Reporting Items for Systematic Reviews and Meta-Analyses (PRISMA) standards. The protocol was published on the PROSPERO database ID: CRD42017083436.

A systematic search of seven databases (MEDLINE, AMED, Cochrane Database of Systematic Reviews, PsychINFO, SPORTDiscus, CINAHL and Web of Science) was performed in September 2017. Search terms were developed in collaboration with an information specialist (Additional file 1). Inclusion and exclusion criteria are listed in Table 1.

**Table 1:** Inclusion and exclusion criteria

Factor	Inclusion criteria	Exclusion criteria
Purpose	Include focus on Sedentary Behaviour (SB) and/or reduction of SB.	Focus on physical activity (PA) but does not explore SB or sedentary time
Sample	Adults (≥18 years), caregivers/friends/family in relation to SB in adults, health care professionals specific to SB in adults. Adults must have had first-hand experience of being sedentary and/or being involved in programmes designed to change SB.	Children or adolescents (≤17 years), caregivers/family/friends in relation to SB in children, paediatric health care professionals Workplace-based studies
Data collection	Primary research studies using qualitative data collection methods for example, study data may be generated through interviews, focus groups, qualitative observational studies	Solely quantitative methods.
Data analysis	Qualitative methodology e.g. thematic, content, framework	Solely quantitative methods. Descriptive accounts where no evidence of qualitative method or analysis
Format	English and peer-reviewed. No date restrictions were applied	Grey literature – posters, conference abstracts, supplements, book chapters, case studies, reviews, dissertations/ thesis, editorials

Following the search, three reviewers (GHR, RW and DJC) jointly screened the first 150 titles and abstracts – this allowed for review and refinement of the inclusion criteria. Thereafter, GHR and RW independently screened the remaining titles and abstracts (50% each). Full text articles were independently reviewed by the same two reviewers; disagreements were resolved through discussion with a third reviewer (DJC). A backward search of references of eligible papers did not identify any additional studies.

### Data extraction

The following data were extracted from each study: author(s), year of publication, study purpose, sample characteristics, country, methodological considerations, findings and discussion. One-third of the included studies were randomly selected and subject to double data extraction and quality assessment. The data extraction tables and quality assessment reports for papers subject to double data extraction and quality assessment were reviewed by a third reviewer (DJC), then discussed with the primary reviewers (GHR and RW). The level of agreement for data extraction was found to be good; there was also a satisfactory level of consistency in the quality assessment ratings for these papers.

### Quality assessment

The National Institute for Health and Care Excellence qualitative appraisal guidance was used to assess methodological quality [17]. Studies were evaluated using a 14-item quality assessment checklist (Table 3). Reviewers endorsed the presence or absence of domain characteristics as clear, unclear or not reported. The checklist assessment of study quality can be marked: (++) the majority of the criteria have been fulfilled; (+) some of the criteria have been fulfilled; or (−) very few of the criteria have been met. Differences in quality assessment ratings between the reviewers were discussed until consensus was reached. Quality was assessed for descriptive purposes; papers were not excluded on the basis of the quality assessment; we drew upon relevant data from all included studies.

### Data synthesis

A thematic synthesis approach was used [18]. Data from primary studies were used to initially develop descriptive themes that closely reflected study findings. Analytical themes were then formulated that go beyond the data and generate new interpretations of the results [18]; this involved three main stages:

1. Key findings, including the title of themes, from each article, specific to the review aims, were coded by GHR and RW using NVivo 10 [19].
2. Codes were organised to identify relationships, similarities and differences between the data. This stage identified key descriptive themes and sub-themes.
3. Analytical themes were developed. This was an iterative and cyclical process. Reviewers explored the descriptive themes to generate novel findings based on the amalgamated data with the view of helping to inform future intervention development, policy and practice towards sedentary behaviour.

In this review, ‘’ represent authors’ quotations whereas “” are used for participants’ own words.

## Results

### Literature search

**Fig. 1:**
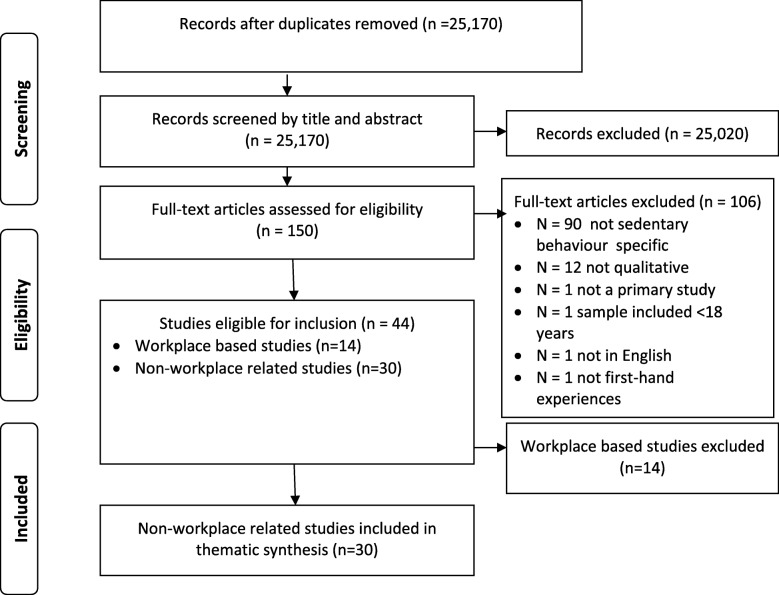
PRISMA diagram

From 25,170 titles and abstracts identified (Fig. 1), 25,020 were excluded. Full texts of 150 papers were assessed for eligibility; 44 were found to be eligible; reasons for exclusions are stated in Fig. 1. The 44 eligible studies fell into two categories; studies of the experiences of individuals outside of the workplace (*n* = 30), including, the experiences of those with a medical condition and those who had participated in programmes to reduce sedentary behaviour and, studies focused on sedentary behaviour in the workplace (*n* = 14). As previously stated, we made a post-hoc decision to remove studies that specifically examined workplace associated sedentary behaviour. Included studies are listed in Table 2. See Fig. 1 for PRISMA diagram and Additional file 2 for the references for the 14 workplace studies.

**Table 2:** Summary of included studies

Primary author and country	Year	Abbreviated aim(s) of study	N and sampling method	Defining participant characteristics (population, gender and age)	Data collection and analysis	Investigating SB intervention	Quality Appraisal
Adams, Gill [37] USA	2015	To investigate feasibility of an intervention aimed at reducing SB in overweight women.	64 Volunteers	Overweight women (BMI > 25) 100% female Mostly aged > 50 yrs.	Open ended questionnaire Inductive analysis	√	+
Ball, Salmon, Giles-corti [43] Australia	2006	To investigate perceived intrapersonal, social and physical environmental influences on PA of women of different SES backgrounds.	56 Snowball	Healthy adults: 19 high-SES, 19 mid-SES and 18 low-SES 100% female Age range 18-65 yrs.	Semi-structured interview Thematic analysis		+
Biddle, Edwardson, Gorely [21] UK	2017	To explore experiences of a workshop to understand outcomes of an intervention aimed at reducing SB in those at risk of type 2 diabetes.	71 Purposive (but unclear)	Adults at risk of type 2 diabetes Data not present for *n* = 71 participants included in qualitative study	Evaluation sheets, progress phone calls, and telephone interview Inductive analysis	√	–
Britten, Addington, Astill [22] UK	2017	To document participant’s views and effects of a dance programme.	22 (but unclear) Purposive	Community dwelling older adults 95% female Mean age 75 yrs	Three focus groups Thematic content analysis	√	+
Chastin, Fitzpatrick, Andrews [31] UK	2014	To investigate the determinants of SB in older adults.	9 Convenience	Older women 100% female Age range 70-92 yrs	Semi-structured interview Framework analysis /thematic analysis approach		++
Chen [29] Taiwan	2010	To explore barriers that older adults experience in PA participation.	90 Purposive	Older adults residing in long-term care 66% female Age range 65-90 yrs.	Interview Content analysis		++
Cousins, Keating [20] Canada	1995	To identify factors to better understanding of life pathways leading women to PA or inactivity.	13 Theoretical	Older women 100% female Age over 60 yrs	Two focus groups consisting of active or inactive women Content analysis		–
Curry, Duda, Thompson [49] UK	2015	To compare perceived PA and ST to objective data, and explore experiences of PA- and ST amongst South Asian women in the UK.	24 Purposive	South Asian women - 92% were either obese or overweight 100% female Age range 36-67 yrs	Semi-structured interview Deductive content analysis		++
Damush, Plue, Bakas [41] USA	2007	To elicit barriers and facilitators of exercise after stroke to inform the development of post-stroke programme.	13 Convenience (but unclear)	Stroke survivors 38% female Mean age 59 yrs	Three focus groups Iterative consensus process		++
Deliens, Deforche, Bourdeaudhuij [47] Belgium	2015	To identify determinants of and recommendations towards PA and SB in Belgian university students.	46 Snowball	University students 63% female Mean age 21 yrs.	Seven focus groups Inductive thematic approach		++
Emadian, Thompson [23] UK	2017	To explore factors influencing PA and ST in overweight or obese South Asian men living in the UK.	31 Purposive	Overweight or obese South Asian men 100% Male Mean age 44 yrs	Semi-structured interview Content analysis		++
Ezeugwu, Garga, Manns [24] Canad	2017	To investigate perceptions of SB in ambulatory stroke survivors.	13 Purposive	Stroke survivor, 46% female Age range 26–75	Semi-structured interview Thematic analysis		++
Grossman, Stewart [33] USA	2003	To explore perceptions, motivations and barriers of PA in underactive community dwelling older adults.	33 Convenience	Older adults 54% female Mean age 80 yrs.	Qualitative interview Standard coding, categorising, indexing, and integration techniques		–
Greenwood-Hickman, Renz, Rosenberg [42] USA	2016	To explore motivators, barriers, and impact of SB reduction among a group of overweight older adults.	24 Convenience	Overweight or obese older adults 67% female Age range 60-84 yrs.	Semi-structured telephone interview Inductive thematic approach	√	++
Guell. Shefer, Griffin [32] UK	2016	To investigate how active living relates to later life experiences, aspirations and strategies of healthy ageing.	27 Purposive	Older adults 44% female Age range 65–80	Semi-structured interviews and observations Thematic analysis		++
Keegan, Middleton, Henderson [48] UK	2016	To identify which socio-environmental factors motivate PA and/or SB, in adults.	15 Stratified	Working-age adults 53% female Age range 31–59	Semi-structured interview Inductive content analysis		++
Kolt, Paterson and Cheung [35] New Zealand	2006	Identify the barriers to PA participation in sedentary older Tongan adults.	24 Snowball	Tongan adults 50% female Age range 60-79 yrs.	Focus groups Descriptive qualitative methodology		++
Leask, Sandlund, Skelton [25] UK	2017	Co-create a tailored public health intervention to reduce SB in older adults.	11 Volunteers	Older adults 55% female Age range 66-82 yrs.	Ten interactive co-creation workshops Qualitative content analysis	√	++
Mabry, Al-Busaidi, Reeves [30] Oman	2013	To identify policy and programme options to address physical inactivity and prolonged sitting in Omani adults.	10 Purposive	Public health managers 50% female	Semi-structured interview Thematic content analysis and a framework approach		++
Martinez-Ramos, Martin-Borras, Trujillo [38] Spain	2015	To examine the opinions of overweight, sedentary patients on ways to reduce this behaviour, their willingness to change, and prospect of receiving help.	23 Convenience	Overweight or obese adults 65% female Age range 25-63 yrs.	Five group and five semi-structured interviews Thematic content analysis		+
Matei, Thune-Boyle, Hamer [34] UK	2015	To explore participant’s views towards an intervention to reduce ST and increase activity in older adults.	35 (but unclear) Purposive	Older adults Aged between 60 and 75	Semi-structured interview Thematic analysis	√	+
McEwan, Tam-Seto, Dogra [26] Belgium	2017	To better understand the perceptions of older adults towards SB.	26 Volunteers	Older adults 77% female Age mean 74 yrs.	Four focus groups and field notes Content analysis		++
Paxton, Anderson, Sakar [39] USA	2016	To identify beliefs, perceptions, and recurrent themes associated with breaking up prolonged periods of sitting.	31 Convenience/Purposive (but unclear)	Breast cancer survivors, 100% female Age range 22- 75 yrs.	Semi-structured telephone interview Content data analysis		++
Shuval, Hebert, Siddiqi [44] USA	2013	To explore impediments and enablers to PA and investigate attitudes toward SB.	25 Purposive	Low income and ethnic minority adults 52% female Age range 30-54 yrs.	Semi-structured interview Framework approach		++
Smetaniuk, Johnson, Creurer [27] Canada	2017	To examine students’ perceptions of factors that influence PA and SB.	43 Convenience	Students in Physical Therapy Age range 22-33 yrs	Photovoice analysis – document, four focus groups Thematic analysis		+
Teychenne, Ball, Salmon [46] Australia	2011	To explore influences on SB in women living in socio-economically disadvantaged neighbourhoods and who are experiencing depressive symptoms.	18 Random	Disadvantaged women experiencing depressive symptoms 100% female Age range 18-46 yrs	Semi-structured telephone interview Thematic analysis		++
Teychenne, Ball, Salmon [45] Australia	2012	To investigate feasibility of two intervention approaches (one print-based and one web- based) designed to promote PA and reduce SB amongst women living in socio-economically disadvantaged areas.	42 Random	Women living in disadvantaged neighbourhoods and key stakeholder 100% female Mean age 50 yrs	Questionnaire Thematic analysis	√	++
Thomsen, Beyer, Aadahl [36] Denmark	2015	To examine how patients with rheumatoid arthritis describe their daily SB.	15 Purposive (but not stated)	People living with rheumatoid arthritis 66% female Age range 23-73 yrs.	Semi-structured interview Thematic analysis		++
Trinh, Arbour- Nicopoulos, Sabiston [40] Canada	2015	To examine perceptions of SB and the preferences for a SB intervention of men on androgen-deprivation therapy.	27 Convenience (but not stated)	Prostate cancer survivors 100% Male Age mean 74 yrs.	Nine focus groups Thematic analysis		++
Van Dyck, Mertens, Cardon [28] Belgium	2017	To examine determinants of PA and SB and needs regarding PA intervention in recently retired adults.	37 Convenience	Recently retired adults 51% female Mean age 63 yrs.	Four focus groups Thematic analysis	√	++

Definition of terms in Table 2: *SB* Sedentary Behaviour, *BMI* Body Mass Index, *PA* Physical Activity, *ST* Sedentary Time, *n* Number,, *SES* Socio-Economic Status, *UK* United Kingdom, *USA* United States of America, *yrs*. Years

### Study characteristics

Studies were published between 1995 [20] and 2017 [21–28]; 25 were published between 2008 and 2017 (Table 2). All but two studies [29, 30] examined the perceptions, experiences and sedentary behaviours of individuals living in Western countries. Whilst contemporary definitions differentiate between sedentary behaviour and physical activity [9], in the papers included in the review, thirteen focused specifically on sedentary behaviour and seventeen considered sedentary behaviour experiences, perceptions or reduction of sedentary behaviour in the context of physical activity participation.

Eleven studies examined experiences and/or perceptions of sedentary behaviour in older adults (typically ≥60 years) [20, 22, 25, 26, 28, 29, 31–35]; ten studies focused on sedentary behaviour in people diagnosed with a medical condition [21, 23, 24, 36–42], four explored the perceived impact of socio-economic status on sedentary behaviour [43–46], two examined university students’ experiences of sedentary behaviour [27, 47], two focused on working-age adults [30, 48], and one focused on cultural influences on sedentary behaviour [49]. The views of 918 individuals from ten countries are represented. Participants’ ages ranged from 18 to 92 years. Sample size ranged from 9 [31] to 90 [29]. In 20 studies, the sample was predominantly female or only recruited females, two studies investigated men only, and the remaining eight studies explored experiences of both men and women.

Overall, 22 studies examined adult’s experiences and perceptions of sedentary behaviour, and eight studies investigated participant’s experiences of engaging in interventions designed to reduce sedentary behaviour. The intervention studies included older adults [22, 25, 28, 34], overweight women [37, 42], women living in disadvantaged neighbourhoods [45] or adults at risk of type 2 diabetes [21].

### Quality assessment

Most studies were graded highly across the fourteen quality domains (Table 3). Twenty-one (70%) papers were graded ++ (good). Six papers were graded + (moderate); in these papers description of data generation and analysis was limited; in five [21, 22, 34, 38, 43] the role of researcher(s) was not described in sufficient detail, and ways in which the relationship between participants and researcher(s) may have influenced the study were not considered. Three papers were rated as - (low). These papers did not clearly report how data were generated, nor the stages of or who was involved in the analysis. These papers did not discuss research limitations and were evaluated as being narrow in their conclusions.

**Table 3:** Methodological quality of studies (numbers refer to the number of studies *n* = 30)

Domain	Rating	
Theoretical rationale: appropriateness	Appropriate	Inappropriate/ Not sure
	29	1
Theoretical rationale: clarity	Clear	Unclear / Mixed
	29	1
Study design	Defensible	Indefensible / Not sure
	28	2
Data collection	Appropriately	Inappropriately / Not sure
	22	8
Trustworthiness: role of researcher	Clear	Unclear / Not described
	18	12
Trustworthiness: clarity	Clear	Unclear / Not sure
	21	9
Trustworthiness: reliability	Reliable	Unreliable / Not sure
	24	6
Analysis: rigorous	Rigorous	Not rigorous / Not sure
	22	8
Analysis: richness	Rich	Poor / Not sure
	25	5
Analysis: reliability	Reliable	Unreliable / Not sure
	22	8
Analysis: convincing	Convincing	Not convincing/ Not sure
	28	2
Analysis: relevance	Relevant	Irrelevant / Partially relevant
	29	1
Conclusions	Adequate	Inadequate / Not sure
	24	6
Ethics	Appropriate	Inappropriate / Not sure
	29	1

To achieve the highest grade (++) consensus between reviewers was required

### Thematic synthesis

**Fig. 2:**
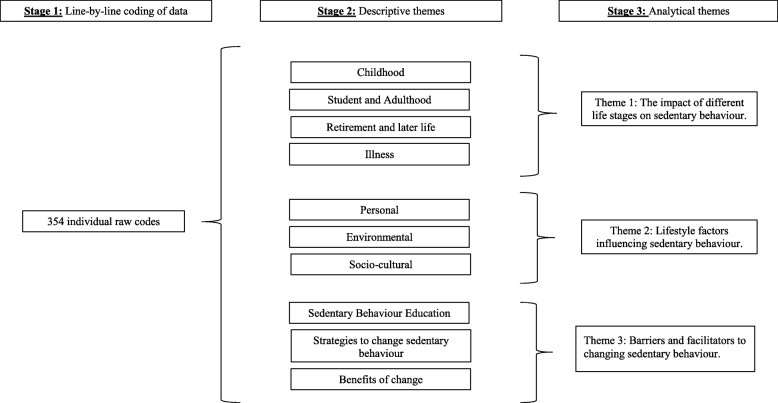
Framework of emergent descriptive and analytical themes

In total, 354 raw codes were recorded, from which ten descriptive themes emerged. After further analysis, three analytical themes were identified focusing on (i) the impact of different life stages on sedentary behaviour, (ii) lifestyle factors influencing sedentary behaviour, and (iii) barriers and facilitators to changing sedentary behaviour (Fig. 2).

### Theme 1: the impact of different life stages on sedentary behaviour

#### Childhood

Some participants perceived their attitudes and behaviour related to their current sedentary behaviours were established in childhood. Individuals explained how social and physical environments in which they grew up in influenced their levels of sedentariness [38, 46]. Parenting style as a determinant of sedentary behaviour was also described. In one study published in 1995 interviewing older women, one ‘*inactive woman*’ [defined as someone who did not exercise ‘*regularly*’] reported that her mother would at times tell her she was “*overdoing it*” and she had “*better sit down and read a book or do a bit of sewing*”. While this reflects a single perspective on the influence of parental attitudes toward activity in a different time period, it highlights the perceived importance of parental influences on shaping later life attitudes toward sedentary behaviour [20].

Other factors impacting levels of sedentary behaviour at this stage were family norms, social pressures, and the interests and capabilities of the participant [20]. For example, a ‘*turning point’* in later childhood was described; individuals would compare, for example, their performance or competency in sport to that of their peers. This comparison led some to focus their efforts on less active pursuits: “*If you are not good at organised sport you are not going to continue it*”. Such turning points could shape later life decisions to engage in pursuits which gave pleasure, such as knitting, needlework and watching television, but which were nonetheless sedentary. [20]. However, attitudes toward sedentary behaviour formed at this stage were not immutable and could be subject to change as a result of later life experiences. In more recent studies, one participant explained that after leaving home her level of sedentary behaviour remained the same as that imposed in the family home [46]*,* while in contrast, another interviewee explained that he was now free to engage in as much sedentary behaviour as he wanted [47]*.*

#### Student and adulthood

Naturally, social and family roles, employment and economic circumstances changed over time in adulthood. Such factors were reported as directly influencing time spent sedentary, the consequences of which, could act as facilitators or barriers to reducing sedentary time. In the two studies focused on university student experiences, students reported engaging in high levels of sedentary behaviour. They identified that academic pressures and university culture required long periods of sitting. The sedentary tendency promoted in academic settings seemed to encroach on other areas of life as participants “*become used to living like that”* [38]. Healthy life choices were described as being “*sacrificed*” over gaining an education. For some, this appeared to be a source of conflict as the behaviour was inconsistent with their knowledge of a healthy lifestyle [27, 47].

Sedentary behaviour associated with employment and the influence of employment on daily life emerged as an important determinant of sedentariness. Employment (or lack of [41]) was described as “*directly*” influencing levels of sedentary behaviour [48]. This was evident across a range of different participant groups. Factors that increased sedentary behaviour included: commuting to work; inconsistent or long working hours meaning people found it difficult to be active; having to sit at a desk; attend meetings [37]; or due to the effects associated with work, including stress and fatigue [38, 43, 48, 49]. In contrast, participants in a study of stroke survivors explained that, after resigning from employment due to their health, they used exercise to fill their empty daily schedules [41].

In adulthood, family roles or “*obligations*” [38] such as increased responsibilities around the home, relationships or being a parent appeared to be a common factor that affected levels of sedentariness: *“you get tied up with the social engagement of your family”* [30]*.* These pressures were also reflected in experiences of sedentary behaviour interventions as family and work commitments were a common barrier to compliance [37, 42]. While physical and time demands associated with children generally limited parents’ opportunities to engage in physical activity, for some, responsibilities for children meant that they did not have the free time to be sedentary. Indeed, some described children as ‘*energetic resources’* [20, 44, 48].

#### Retirement and later life

Older participants, in the later stages of life, described a general slowing down and becoming more sedentary as a result of internal (i.e. interests, routines and ageing) and external (i.e. expectations, social norms) pressures. The hobbies and leisure activities that older adults took part in were predominately sedentary e.g. passive television (TV) viewing [25, 28], reading, sewing [38], and knitting [26]. It seemed that while older adults acknowledged the negative consequences related to their sedentary activities, such concerns were displaced if the behaviours were enjoyable, and associated with cognitive or social benefits: ‘*Many of the participants described how their preferred sedentary behaviour provided them opportunities to meet new people*’ [22].

Stigmatising aspects of participants’ social identities also emerged and cohered around the view that older adults can be viewed by others as ‘*tired, sick, lonely, or depressed’* [26], and that they should ‘*sit all day’*. While it was not explicitly reported, this view appeared to be held by society, friends and family (as well as some older adults themselves). Older adults interviewed in one study reported feeling ‘*typecast*’ as “*not useful*” or “*unable*” and that sitting should be their *‘main mode of living’* [31]. Despite these perceived pressures, some participants endeavoured to stay physically active, and harboured what was described as an ‘*active ageing attitude’* [32]*.* Notwithstanding this however, older adults’ experiences and perceptions of limitations in relation to their ageing bodies appeared highly salient: “*I use to do a lot more things but now… you just can’t do it*” [26, 29]. For some, an increase in sedentary behaviour was motivated by their concerns that ‘*standing up more would interfere with the strategies they had put in place’,* in response to their declining health or mobility [31] (see 3.5.4).

There were mixed views about the health benefits of reducing sedentary behaviour and maintaining a physically active lifestyle. A widely held belief that older adults should ‘*rest’* [31] was reported, and whilst encouraging rest may be perceived as a ‘*caring gesture’* by family or friends, participants pointed out that this behaviour ‘*took opportunities for being active and independence away from them’* [31]. On the other hand, some studies highlighted how family members positively influenced and supported older adults to reduce time spent sedentary through the shared responsibility of looking after grandchildren [48, 49].

#### Illness

It was commonly reported that the impact of poor health contributed to prolonged periods of sedentary behaviour. Participants explained that symptoms associated with health status, such as *‘fatigue’* and *‘pain’* increased sedentary behaviour [24, 36, 39]. Interestingly however, pain and stiffness were also reported as reasons for breaking up periods of sedentary behaviour and increasing activity levels [40–42]. This bi-directional relationship between sedentary behaviour and illness was further exemplified when participants described sedentariness and mental health [31, 38]. Depression was commonly linked to use of sedentary behaviour [24, 31] with some explaining that: ‘*overcoming depression is essential to reducing sedentary behaviour’* [24], that they became more active ‘*to fight depression’* [31], or sedentary behaviour was used to ‘*switch off’* and ‘*remove themselves from their depressive frame of mind’* [46].

Engaging in sedentary behaviour was a common strategy used by participants to prevent declining health or further injury, and transitioning back into illness. Sedentary behaviour was adopted by some as a means to recover from and manage chronic disease symptoms [24, 31] and rest was viewed as an important element in the recovery process [24], suggesting that sedentary behaviour was used as a precautionary or protective behaviour. There was also evidence to suggest that caring for and looking after family or friends who lived with a health problem reduced levels of activity and increased sedentary time [31, 36, 38, 48]: *“My wife has a serious lung disease. We are very limited in doing things …Before, we always went out to concerts”* [36]*.*

### Theme 2: lifestyle factors influencing sedentary behaviour

#### Individual

The range of sedentary behaviours individuals reported engaging in were considerable and included: reading, watching TV, crosswords, meditation, knitting, bingo, eating, gaming, studying, religious functions, motorised transport and ‘*simply lying down’* [24]. Participants’ interests (or at least their levels of activity and sedentary behaviour [30]) seemed to be influenced by age, gender [48], physical mobility, culture [49] and socio-economic status [43]. One interviewee reported that the sedentary activities engaged in ‘*were an important part of their life and self-image’* [42] and to change this would not only be difficult, but it would change who they are as a person. Participants in two studies reported engaging in sedentary behaviour because it was “*comfortable*” and “*relaxing*” [24, 38]. Indeed, this was described as a potential barrier as people were concerned that breaking up sedentary behaviour would ‘*ruin*’ their enjoyment. In line with the pleasurable attributes of sedentary behaviour, individuals described using it as a reward [42]. Given this level of enjoyment, certain sedentary activities appeared to be a *‘compulsion’* as some participants described needing ‘*self-discipline’* [47] or having to make a ‘*conscious effort’* [24] to be less sedentary.

People reported engaging in sedentary behaviour for specific activities, such as reading or using the computer. This was due to the associative benefits, for example, when engaged in sitting individuals reported that they could give greater attention to the task at hand [25]. Sedentary behaviour however was not always associated with interests, need or comfort as it was also attributed to people being ‘*lazy*’, using it to ‘*pass the time’* [46] or their disinterest in more active pursuits [31, 36]. As such, sedentary behaviour appeared to be an “*easy*”, [32, 42] cheap or habitual alternative [47] to more active behaviours. Some forms of sedentary behaviour were seen as being integral components to daily life [25], for instance, across studies it was common for participants to sit down to rest after work [31, 38, 48, 49]. However, participants involved in focus groups investigating their experiences of a sedentary behaviour intervention explained that, reducing sitting time at home or in the evenings would be easier than limiting sitting at work [21].

There were a number of facilitators towards individuals being more active (and reducing sedentary behaviour). These included: being ‘*motivated and determined’* to be less sedentary [24, 48]; adhering to physical activity guidelines; motivated to age well [32]; to keep their independence [33]; and to look good and be healthy.

#### Environmental

Individuals’ physical environment was an important factor when understanding the determinants of sedentary behaviour. People described being more likely to be sedentary during the winter months, when it was cold or wet, and short daylight hours [23, 24, 26, 31, 38, 40, 45, 46]. In line with this, symptoms associated with illnesses or ageing were barriers, for example, people with impaired eyesight expressed concerns over obstacles i.e. shrubs, which posed as hazards [33].

Other practical constraints influencing sedentariness were financial costs [22], poor transport links making walking to certain places difficult; home location [48], work-life balance and neighbourhood crime. Problems associated with childcare [46], and lack of availability of gyms, parks or greenspace, and poor quality of services [21, 44, 46] were also reported. Similar restrictions were described as logistical barriers by participants in sedentary behaviour interventions [22]. There was evidence to suggest that some individuals externalised fault, blaming practical factors for being less sedentary; when students were asked how they could reduce their sedentary behaviour, they predominately reported changes that others could make as opposed to actions that they could perform themselves [27, 47].

#### Socio-cultural

Family, friends and pets [27, 32, 38, 41] were described as being able to prompt, remind and motivate participants to decrease their sitting time and engage in more physically active pursuits [42, 46, 48]: “*He [a friend] lost three stone in a year…And it suddenly clicked and I decided I wasn’t a lost cause*” [48]. However, they could also discourage participants: one interviewee explained that if she went out on a Saturday with her mother she would “*go on foot*”, whereas if she went out with her father they would go by car as he “*doesn’t want to walk*” [38]. The benefits of social support were also described by participants in sedentary behaviour interventions, such as meeting new people, or feeling that they must attend sessions as to not let others down [22]. Although the current review included only two studies examining sedentary behaviour outside of Western culture, different socio-cultural norms and family traditions were shown to influence sedentary behaviour [42]. For example, in one study examining sedentary behaviour in South Asian women living in the United Kingdom (UK), the culturally accepted norm when becoming a mother-in-law was being ‘*entitled to do a great deal of sitting after having raised a family*’ [49].

Notwithstanding a high proportion of the studies reviewed here examined a female dominant sample, a strong gendered dimension emerged [30, 43, 48]. Several participants made reference to the limited culturally appropriate options to be less sedentary available to women: “*The ladies who have no job, what [option] will they have except sitting at home? They cannot just go around roaming between the houses, socially it’s not acceptable*” [30]. Differences in socio-economic status appeared in the value afforded to certain leisure-time sedentary behaviours. Women of all socio-economic groups reported preference for TV viewing, but this appeared particularly popular as a pastime among women of low socio-economic status and, to a lesser extent, mid socio-economic status [43, 46].

The media reportedly played an important role in influencing participants’ perceptions of sedentariness. While it helped some individuals to live a healthier lifestyle, for others, it desensitised them or caused feelings of hopelessness as they felt there is little they could do about being sedentary [26, 48]. The importance of how key messages around sedentary behaviour are delivered was further demonstrated in intervention studies, as participants explained some of the information provided came across as being patronising [42, 45].

### Theme 3: barriers and facilitators to changing sedentary behaviour

#### Sedentary behaviour education

Many participants were unfamiliar with the term sedentary behaviour and were not aware of the associated health consequences [23–25, 28, 47, 49]. Further, misconceptions around sedentary behaviour were described: a stroke survivor showed ‘*surprise when told that lying down during non-sleeping hours was considered sedentary behaviour*’ [24]. Lack of knowledge contributed to cognitive distortions with some individuals demonstrating all-or-nothing thinking, perceiving that if they were not physically active they must be sedentary [21, 46, 47]. Other participants found it difficult to understand that their level of sedentary behaviour was problematic because they regularly engaged in physical activity [42]. Also, as discussed in Theme 1 (retirement and later life), there seemed to be cognitive dissonance around sedentary behaviour as while many viewed sedentariness negatively, they felt that the seated activities they engaged in were not negative because they perceived those behaviours had ‘*many social and cognitive benefits’* [26]. Despite participants’ limited knowledge of sedentary behaviour, it was apparent that, on some level, individuals did understand that living a sedentary lifestyle was unhealthy. For example, participants described the guilt they associated with being sedentary [38, 46], negative connotations and the stigma of identifying as being sedentary [26, 48]; and some actively reduced their sedentary behaviour to be a good role model [46]. In one intervention study, participants described the link between too much sitting and health as ‘*logical, maybe even obvious*…’ [21].

Educating people about sedentary behaviour was a common suggestion made by participants and researchers to reduce sedentary time. Participants felt this could be achieved in schools, workplace settings, community centres, places of worship, and health and social care settings [23, 38]. Although none of the studies included in this review explored the perceptions or experiences of healthcare professionals in relation to sedentary behaviour, healthcare providers reportedly played an important role in educating and influencing participants’ sedentary behaviour. One interviewee explained [48, 49]: “*I actually do stand a lot when I’m watching TV…I’ve been given advice by my GP [General Practitioner] to do it*” [46].

#### Strategies to change sedentary behaviour

Different strategies were described to reduce the total amount and break up bouts of sedentary behaviour. In one study [21] participants were asked to list key strategies used to ‘*sit less or move more*’ during a sedentary behaviour intervention. Eighteen different methods were suggested; the most common being walking, standing during TV breaks, reducing or turning off the TV, going to the gym, and standing while talking on the phone [21]. However, participants tended to focus more on strategies that specifically increased physical activity providing ‘*little to no specific recommendations’* targeting sedentary behaviour [21, 46, 47] [46]. In intervention studies, there was a mixture of attitudes towards alternatives to sedentariness. Some participants were not in favour of modifying current sedentary behaviour, doubted the effectiveness of suggested strategies [21] or felt alternatives were too artificial or forced [42]. Others however, appeared to enjoy this version of ‘*multitasking*’ as it was a ‘*new way of exercising’* [22]. Nevertheless, it was clear that any changes needed to be incorporated into participants’ everyday lives and become habitual [34].

Participants in sedentary behaviour interventions described the use of different behaviour change techniques. These included: monitoring their own sedentary behaviour [42]; having the opportunity to problem solve and overcome barriers to being more active; reading leaflets or booklets that discuss the importance of physical activity and reducing sitting time [45]; and regular prompts and reminders, for instance, key messages such as *‘sit less’, ‘move more’* and ‘*stand more’* [21]). Financial incentives (e.g. reduced gym fees); opportunities for social comparisons and support [21]; being able to set their own sedentary reduction goals [25, 42]; praise from others [42]; and rewards for reducing sedentary behaviours [40] were also reported. Technology-related behaviour change techniques were discussed including, wearable devices and computer or smart-phone/tablet applications (apps). Such strategies were described as helping to track progress, ‘*enable*’, ‘*prompt*’ or ‘*remind*’ participants to sit less [21], as well as being a key resource for information. While many participants had something positive to report about these methods, problems were experienced - this typically consisted of devices not being user friendly or practical [21, 42].

For some people, their experience of the strategies designed to alter sedentary behaviour seemed to change as the intervention progressed. It was noted, for example, that some techniques could become rather agitating or frustrating and some individuals felt that failing to achieve intervention goals “*could be depressing*” [45]. Both substantial, long-term changes as well as more subtle, short-term nudges to reduce sedentary behaviour were suggested [21, 45]. It was identified that strategies to reduce sedentary behaviour had to be suitable, straightforward, achievable, enjoyable [35, 37], time efficient, and tailored to the individual’s particular circumstance, ability, and personal characteristics (such as age or gender) [45]. Indeed, ‘*the suitability of the activities could either motivate physical activity or sedentariness’* [48]; in one study, stroke survivors explained that if strategies were unsustainable or unrealistic, then they were ‘*needless*’ [24].

#### Benefits of changing sedentary behaviour

Through changing levels of sedentary time and activity, participants in sedentary behaviour intervention studies reported a range of benefits. This included: increased stamina; balance; weight loss [21]; general ‘*physical and psychological’* wellbeing [22]; a more active and ‘*fulfilling*’ life; pride at having made a change [42]; improved mood; enhanced sleep quality [34]; cognitive benefits; quality of life and ‘*mental health’*. Participants explained that they were motivated to change by the short-term achievements “…*you’re immediately rewarded when you stand up and you’re not so stiff…*” [42]) as well as the anticipated long-term gains [25, 42]: “*Weight loss always motivates women*” [45].

## Discussion

This review aimed to synthesise current knowledge in regards to the experience and perception of sedentary behaviour and participation in interventions designed to reduce sedentary behaviour in adults. We synthesised data from 918 participants from 30 studies and identified three analytical themes: (i) the impact of different life stages on sedentary behaviour, (ii) lifestyle factors influencing sedentary behaviour and (iii) barriers and facilitators to changing sedentary behaviour.

The first theme reflected the micro and macro pressures experienced at different life stages that are influential in shaping individuals’ beliefs, attitudes and behaviour related to sedentariness. The Capability, Opportunity, Motivation and Behaviour (COM-B) model [50] recognises that behaviour is part of an interacting system. The heterogeneous nature of the participant groups in the current review allowed us to trace how these different components may be shaped depending on life stage. In childhood, individuals described having the motivation and capability of being active; however, parental and academic influences could limit opportunities, sometimes promoting sitting time. In adulthood, all components were influenced by personal experiences, social and working commitments, and economic circumstances. Overall, in the studies reviewed, this meant that participation in exercise reduced and sedentariness typically increased between childhood and adulthood. In later life, declining health meant that individuals were not always capable of being active and cultural expectations reduced opportunities, promoting sedentariness, regardless of whether individuals were motivated to be less sedentary or not.

Participants in some studies described using sedentary behaviour to cope with changes in health status. Increased sedentary behaviour in illness has been reported elsewhere [51]. Notwithstanding that some sedentariness is necessary and inevitable in illness; our review highlights other important motivations behind this behaviour, suggesting it is also perpetuated by social and family norms, personal experiences and associated benefits, such as gratification. There is a risk however, that using sedentary time as a protective behaviour could become a self-fulfilling prophecy. For example, the belief that sedentary behaviour must be engaged in when ill, in addition to declining physical fitness caused by limited activity, may lead to further reduced mobility and impact negatively on health. Additionally, this behaviour may be generalised to cope with other demands associated with daily life.

Interventions designed to reduce sedentary behaviour should consider external and internal influences on individuals and groups at different life stages [52]. Individuals with (and without) medical conditions may need specific support to develop alternative coping techniques associated with less health risk.

The second theme demonstrated the multifaceted nature of sedentary behaviour. In our review, sedentary behaviour reportedly played a large role in participants’ daily lives. However, the motives behind the adoption of this behaviour differed. When looking to change behaviour it is important to first formulate and understand the behaviour and approach the situation in a balanced way, recognising that not all sedentary behaviours/activities are inherently negative. Identifying personalised goals for sedentary behaviour reduction [53] will help guide what and how intensive behaviour change strategies need to be. This can incorporate understanding core beliefs associated with sedentary behaviours and identify alternatives to and adapt existing sedentary activities.

Environmental factors, in particular the weather, were commonly discussed as variables influencing sedentariness. The environmental barriers were similar to those reported in the literature on physical activity [54]. To reduce many of the practical barriers, sedentary behaviour reduction interventions could target where and when to change behaviour; while exercise is likely to be managed externally (away from the home), reducing sedentary behaviour can be achieved in the workplace or at home.

The third theme identified that while physical activity appears to be a widely understood term, the concept of sedentary behaviour and associated negative health consequences were less well known. Moreover, some participants dichotomised sedentariness and physical activity, believing that, if they are not physically active in line with guidelines they are sedentary, thus failing to recognise the value of light intensity physical activity as means to reduce sedentary behaviour. There is a need to educate people about the health risks of sedentary behaviour, as well as about methods and benefits of reducing sedentariness. However, Leask et al. pointed out that people are unlikely to be motivated to reduce their time spent sedentary if they are unaware or do not understand the impact of sedentary behaviour. Moreover, due to the importance and enjoyment of sedentary-based activities, ‘*demonising*’ all forms of sedentary behaviour is unlikely to be effective [25]. A sedentary behaviour reduction programme co-produced by older adult’s highlighted the value of adequately and sensitively framing this kind of information. Group members suggested educational approaches should focus on the ‘*drawbacks*’ of sedentary behaviour as well as the positives of reducing sedentary behaviour and emphasise that some sedentary behaviours are ‘*beneficial*’, such as cognitively stimulating seated activities [25]. Making a distinction between active, purposeful and passive sedentary activities is likely to be beneficial; this categorisation is consistent with how some individuals conceptualise and justify their sedentary behaviour [55]. In addition, given that some participants recognised the negative effects of sedentary behaviour and yet were still sedentary, it is clear that knowledge alone is insufficient to bridge the gap between cognitions and behaviour or to bring about sustained change. Additional strategies are required that look to serve different functions. Education may be effective in managing beliefs about sedentary behaviour. However, other methods such as individually tailored goal setting and action planning are needed to change established behaviours. Strategies aimed at initiating change will not necessarily be sustainable and methods to maintain change are unlikely to be acceptable if initial strategies fail to motivate individuals. Although we have identified some of the motivators for reducing sedentary behaviour, we are unable to draw firm conclusions concerning which sedentary behaviour specific strategies could be implemented and for what populations. Our findings do however support the use of multiple techniques and intervention functions [50], and confirm that one single approach is unlikely to be suitable for all.

In highlighting the multifaceted nature of sedentary behaviour, our review findings are consistent with the elements of the social-ecological model [56] and also with the findings of a consensus study that developed a system-based framework consisting of six clusters of determinants of sedentary behaviour [57]. Sedentary behaviour in adults is influenced by a range of interrelated factors; public health interventions must take account of these factors. Strategies to reduce sedentary behaviours must be easily incorporated into participants’ daily lives and be purposeful.

### Limitations

We only included studies published in English and the majority of studies reviewed examined experiences of sedentary behaviour in Western countries. Therefore, the review findings cannot easily be generalised to other parts of the world. Exploring experiences of sedentary behaviour in a range of different cultures and populations would provide further insight into how socio-cultural, socio-economic and environmental factors shape peoples’ attitudes and behaviours towards sedentariness.

There is no single, best approach to conduct a qualitative synthesis. Instead, the method used should be guided by the aims and purpose of the synthesis [58, 59]. We used a thematic synthesis approach in this review; but we recognise that there is debate about whether it is appropriate to synthesis data generated in research using different qualitative methods. One limitation of qualitative synthesis such is that the meta-themes developed are often broad and overarching; the specific contexts in and about which participants speak are difficult to retain in this kind of synthesis.

An additional aim of the review and thematic synthesis was to explore the views of carers, relatives and health and social care professionals in relation to sedentary behaviour in adults. We did not find any articles investigating views of these groups in relation to sedentary behaviour. It is possible that relevant articles were missed because our search terms were not specific to carers, relatives and health and social care professionals. Research is needed to the explore role that carers, relatives and health and social care professionals play in influencing sedentariness and whether and how their roles can be optimised.

The initial search for this review was conducted at the end of September 2017. We acknowledge that this area of public health research is experiencing considerable growth in numbers of publications. Studies since the end of September 2017 were not included in the current synthesis. Recognising this limitation, we repeated the search using the same parameters in April 2019. Overall, 7273 unique articles were identified. Two reviewers completed title and abstract screening and identified 33 titles for full text screening; nine of these studies met our criteria. Five papers investigated sedentary behaviour in those with a medical condition [60–64], three explored factors affecting older adults’ sedentary behaviours and the acceptability of potential strategies to reduce sedentary time [65–67] and one focused on factors influencing time spent in sedentary behaviour and explored strategies to reduce this sedentariness in African American women in home, work, and social environments [68]. This demonstrates the growing interest in understanding people’s experiences of sedentary behaviour. Whilst the reported findings of these studies appear to be largely consistent with those we report following our thematic synthesis, the iterative nature of a thematic synthesis means that it would not have been appropriate to analyse and interpret these data in a post-hoc addition to our synthesis. What is more, qualitative research is less concerned with generalisability of findings, as it is with seeking situational, as opposed to demographic representativeness [69].

## Conclusions

Sedentary behaviour is influenced by a complex interaction between individual, environmental, socio-economic and socio-cultural factors. Micro and macro pressures are experienced at different life stages, including childhood, adulthood, and later-life and in the context of long-term illness that shape individuals’ beliefs and behaviour related to sedentariness. Our findings suggest that knowledge of sedentary behaviour and the associated health consequences is limited in adult populations. At a population level there is a need for a clear and understandable definition of sedentary behaviour. This should be associated with provision of accessible information about ways in which sedentary behaviour reduction might be integrated in peoples’ daily lives. Interventions targeting a reduction in sedentary behaviour will need to consider the multiple influences on sedentariness when designing and implementing interventions.

## Additional files

Additional file 1: Example search strategy (MEDLINE) (DOC 25 kb)

Additional file 2: Excluded workplace-based studies (DOC 56 kb)

## Data Availability

Not applicable.

## Abbreviations

Apps: Applications
BMI: Body Mass Index
COM-B: Capability Opportunity, Motivation and Behaviour
GP: General Practitioner
N: Number
NIHR: National Institute for Health Research
PA: Physical Activity
PGfAR: Programme Grants for Applied Health Research
PRISMA: Preferred Reporting Items for Systematic Reviews and Meta-Analyses
SB: Sedentary Behaviour
SES: Socio-Economic Status
ST: Sedentary Time
TV: Television
UK: United Kingdom, USA United States of America
yrs: Years
