# Supplementary material for: Exploring adults’ experiences of sedentary behaviour and participation in non-workplace interventions designed to reduce sedentary behaviour: a thematic synthesis of qualitative studies
Source: BMC Public Health. 2019 Aug 13;19:1099. doi: 10.1186/s12889-019-7365-1 (PMC6692932; doi:10.1186/s12889-019-7365-1)
Supplement: Supplementary file 1 — Example search strategy (MEDLINE) (DOC 25 kb) [file 12889_2019_7365_MOESM1_ESM.doc]

Database: Ovid MEDLINE(R) <1946 to September Week 3 2017>

Search Strategy:

--------------------------------------------------------------------------------

1 Sedentary Lifestyle/ (6399)

2 (sedentary or sitting or sedentariness).ti. (5813)

3 ((sedentary or sitting or seated) adj5 (behavio* or lifestyle or life-style)).tw. (6368)

4 (sedentary adj3 (adult? or men or women or males or females or individuals or people or population?)).tw. (4832)

5 ((sitting or sit or seated or stationary or standing) adj3 (task* or time or bout* or work* or break*)).tw. (4195)

6 passive standing.tw. (44)

7 low energy expenditure.tw. (137)

8 physical* inactiv*.tw. (6123)

9 (leisure time adj5 (physical* activ* or passive or inactiv*)).tw. (3194)

10 "physical activity level*".tw. (5926)

11 ((sitting or lying) adj2 posture*).tw. (940)

12 (prolong* adj2 (reclin* or sit or sitting or seated)).tw. (480)

13 "couch potato".tw. (39)

14 (nonexercis* or non exercis* or no exercis*).tw. (3324)

15 chair rise?.tw. (292)

16 "sit* less".tw. (582)

17 ((light or low) adj "physical activ*").tw. (1621)

18 ((decrease or reduc* or discourag* or lessen*) adj3 (sit or sitting or stand or standing or physical* inactiv*)).tw. (1307)

19 (time adj5 (computer* or television or tv or video game? or videogame? or gaming or screen or media)).tw. (8476)

20 ((watch* or view*) adj5 (television or tv)).tw. (4018)

21 (play* adj5 (video game? or videogame? or computer game?)).tw. (1232)

22 or/1-21 [sendentary behaviours] (49252)

23 ((chair or car or automobile or auto or bus or "motor transport" or train or indoor or in-door or commut* or driving) adj3 time).tw. (1939)

24 (physical environment* or built environment or indoor* or "non screen" or passive transport*).tw. (26365)

25 (computer* or television or tv or video game* or videogame* or gaming).ti. (71783)

26 or/23-25 [specific terms] (99823)

27 (sedentary or sit or sitting or seated or inactiv* or underactiv* or under activ*).tw. (316852)

28 26 and 27 [specific terms and sedentary behaviour] (1434)

29 22 or 28 [all sedentary terms] (49892)

30 qualitative.tw. (148688)

31 interview/ (27524)

32 narrative*.tw. (21693)

33 (personal adj2 experience*).tw. (13227)

34 interview*.ti. (30699)

35 exp qualitative research/ (36222)

36 (survey* or questionnaire* or "focus group*").tw. (780917)

37 theme*.ti. (3133)

38 "Surveys and Questionnaires"/ (396996)

39 Focus Groups/ (24032)

40 or/30-39 (1112001)

41 exp animals/ not humans.sh. (4578683)

42 40 not 41 [qualitative filter] (1065935)

43 29 and 42 [sendentary and qualiative filter] (13307)

44 (exp adolescent/ or exp child/ or exp newborn/) not ((exp adult/ or exp aged/ or exp middle aged/) and (exp adolescent/ or exp child/ or exp newborn/)) (1657010)

45 43 not 44 [Adult only studies] (10322)
