# Supplementary material for: Exploring adults’ experiences of sedentary behaviour and participation in non-workplace interventions designed to reduce sedentary behaviour: a thematic synthesis of qualitative studies
Source: BMC Public Health. 2019 Aug 13;19:1099. doi: 10.1186/s12889-019-7365-1 (PMC6692932; doi:10.1186/s12889-019-7365-1)
Supplement: Supplementary file 2 — Excluded workplace-based studies (DOC 56 kb) [file 12889_2019_7365_MOESM2_ESM.doc]

Supplement 2 Summary of excluded workplace studies

| Primary author and year published | Title |
| --- | --- |
| (Ahtinen et al., 2016) | Walk as you work - User study and design implications for mobile walking meetings |
| (Chau et al., 2014) | Desk- based workers' perspectives on using sit- stand workstations: a qualitative analysis of the Stand@Work study. |
| (Cole et al., 2015) | "They should stay at their desk until the works done": a qualitative study examining perceptions of sedentary behaviour in a desk-based occupational setting |
| (De Cocker et al., 2015) | Acceptability and feasibility of potential intervention strategies for influencing sedentary time at work: focus group interviews in executives and employees. |
| (Dutta et al., 2015) | Experience of switching from a traditional sitting workstation to a sit-stand workstation in sedentary office workers. |
| (George et al., 2014) | Physical Activity and Sedentary Time: Male Perceptions in a University Work Environment |
| (Gilson et al., 2011) | Occupational sitting time: employees' perceptions of health risks and intervention strategies |
| (Gilson et al., 2012) | Occupational sitting: practitioner perceptions of health risks, intervention strategies and influences |
| (Graves et al., 2015) | Evaluation of sit- stand workstations in an office setting: A randomised controlled trial. |
| (Hadgraft et al., 2017) | Reducing occupational sitting: Workers perspectives on participation in a multi-component intervention |
| (Löffler et al., 2015) | Office Ergonomics Driven by Contextual Design |
| (Taylor et al., 2013) | Booster Breaks in the workplace: participants’ perspectives on health- promoting work breaks. |
| (Torbeyns et al., 2017) | The potential of bike desks to reduce sedentary time in the office: a mixed-method study. |
| (Waters et al., 2016) | Assessing and understanding sedentary behaviour in office- based working adults: a mixed-method approach |

AHTINEN, A., ANDREJEFF, E., VUOLLE, M. & VÄÄNÄNEN, K. 2016. Walk as you work - User study and design implications for mobile walking meetings. In: Paper presented at the 9th Nordic conference on human-computer interaction. Gothenburg: Wiley; 2016.

CHAU, J., DALEY, M., SRINIVASAN, A., DUNN, S., BAUMAN, A. & VAN DER PLOEG, H. 2014. Desk- based workers' perspectives on using sit- stand workstations: a qualitative analysis of the Stand@Work study. *BMC Public Health,* 14**,** 752.

COLE, J., TULLY, M. & CUPPLES, M. 2015. "They should stay at their desk until the works done": a qualitative study examining perceptions of sedentary behaviour in a desk-based occupational setting. *BMC Research Notes,* 8.

DE COCKER, K., VELDEMAN, C., DE BACQUER, D., BRAECKMAN, L., OWEN, N., CARDON, G. & DE BOURDEAUDHUIJ, I. 2015. Acceptability and feasibility of potential intervention strategies for influencing sedentary time at work: focus group interviews in executives and employees. *Int. J. Behav. Nutr. Phys. Act.,* 12.

DUTTA, N., WALTON, T. & PEREIRA, M. 2015. Experience of switching from a traditional sitting workstation to a sit-stand workstation in sedentary office workers. *Work,* 52**,** 83.

GEORGE, E. S., KOLT, G. S., ROSENKRANZ, R. R. & GUAGLIANO, J. M. 2014. Physical Activity and Sedentary Time: Male Perceptions in a University Work Environment. *American Journal of Men's Health,* 8**,** 148-158.

GILSON, N., STRAKER, L. & PARRY, S. 2012. Occupational sitting: practitioner perceptions of health risks, intervention strategies and influences. *Health Promotion Journal of Australia,* 23**,** 208-212.

GILSON, N. D., BURTON, N. W., UFFELEN, J. G. Z. & BROWN, W. J. 2011. Occupational sitting time: employees' perceptions of health risks and intervention strategies. *Health Promotion Journal of Australia,* 22**,** 38-43.

GRAVES, L. E. F., MURPHY, R. C., SHEPHERD, S. O., CABOT, J. & HOPKINS, N. D. 2015. Evaluation of sit- stand workstations in an office setting: A randomised controlled trial. *BMC Public Health,* 15**,** <xocs:firstpage xmlns:xocs=""/>.

HADGRAFT, N., WILLENBERG, L., LAMONTAGNE, A., MALKOSKI, K., DUNSTAN, D., HEALY, G., MOODIE, M., EAKIN, E., OWEN, N. & LAWLER, S. 2017. Reducing occupational sitting: Workers perspectives on participation in a multi-component intervention. *International Journal of Behavioral Nutrition and Physical Activity,* 14.

LÖFFLER, D., WALLMANN-SPERLICH, B., WAN, J., KNÖTT, J., VOGEL, A. & HURTIENNE, J. 2015. Office Ergonomics Driven by Contextual Design. *Ergonomics in Design: The Quarterly of Human Factors Applications,* 23**,** 31-35.

TAYLOR, W. C., KING, K. E., SHEGOG, R., PAXTON, R. J., EVANS-HUDNALL, G. L., REMPEL, D. M., CHEN, V. & YANCEY, A. K. 2013. Booster Breaks in the workplace: participants’ perspectives on health- promoting work breaks. *Health Education Research,* 28**,** 414-425.

TORBEYNS, T., DE GEUS, B., BAILEY, S., DECROIX, L. & MEEUSEN, R. 2017. The potential of bike desks to reduce sedentary time in the office: a mixed-method study. *Public Health,* 144**,** 16-22.

WATERS, C., ER, P., CHU, A., NG, S., CHIA, A. & MULLER-RIEMENSCHNEIDER, F. 2016. Assessing and understanding sedentary behaviour in office- based working adults: a mixed-method approach. *BMC Public Health,* 16.
